# Polyamine Metabolism and Gene Methylation in Conjunction with One-Carbon Metabolism

**DOI:** 10.3390/ijms19103106

**Published:** 2018-10-10

**Authors:** Kuniyasu Soda

**Affiliations:** Cardiovascular Research Institute, Saitama Medical Center, Jichi Medical University, 1-847 Amanuma, Omiya, Saitama-city, Saitama Prefecture 330-8503, Japan; soda@jichi.ac.jp; Tel.: +81-48-647-2111

**Keywords:** polyamine, spermine, spermidine, methylation, DNA, lymphocyte function-associated antigen 1 (LFA-1), LFA-1 promoter (ITGAL), one carbon metabolism, DNA methyltransferases (DNMT)

## Abstract

Recent investigations have revealed that changes in DNA methylation status play an important role in aging-associated pathologies and lifespan. The methylation of DNA is regulated by DNA methyltransferases (DNMT1, DNMT3a, and DNMT3b) in the presence of *S*-adenosylmethionine (SAM), which serves as a methyl group donor. Increased availability of SAM enhances DNMT activity, while its metabolites, *S*-adenosyl-l-homocysteine (SAH) and decarboxylated *S*-adenosylmethionine (dcSAM), act to inhibit DNMT activity. SAH, which is converted from SAM by adding a methyl group to cytosine residues in DNA, is an intermediate precursor of homocysteine. dcSAM, converted from SAM by the enzymatic activity of adenosylmethionine decarboxylase, provides an aminopropyl group to synthesize the polyamines spermine and spermidine. Increased homocysteine levels are a significant risk factor for the development of a wide range of conditions, including cardiovascular diseases. However, successful homocysteine-lowering treatment by vitamins (B6, B12, and folate) failed to improve these conditions. Long-term increased polyamine intake elevated blood spermine levels and inhibited aging-associated pathologies in mice and humans. Spermine reversed changes (increased dcSAM, decreased DNMT activity, aberrant DNA methylation, and proinflammatory status) induced by the inhibition of ornithine decarboxylase. The relation between polyamine metabolism, one-carbon metabolism, DNA methylation, and the biological mechanism of spermine-induced lifespan extension is discussed.

## 1. Introduction

Aging is associated with declines in physiological function, altered immune function, increased proinflammatory status, and increased susceptibility to pathologies such as cardiovascular disease, cancer, sarcopenia, and metabolic and neurodegenerative diseases. The prevalence of aging-associated diseases and life-span ranges widely, even in countries and areas with similar social and economic conditions. For example, among European and Western countries, there are significant differences in both life expectancy at birth and the incidence of cardiovascular diseases (CVDs), one of the most frequent aging-associated conditions. There is also a close inverse correlation between life expectancy and age-standardized mortality rates due to CVDs [1]. Epidemiological analyses and interventional trials have shown that differences in food preferences and dietary patterns are among the many life-style factors that may play a role in the inhibition of aging-associated diseases and senescence. For example, increased consumption of soybeans and their byproducts is associated with a decreased incidence of CVDs [2,3] and malignancies such as breast [4,5,6] and colon cancer [7,8,9,10]. A mediterranean diet and increased vegetable intake are also associated with a decreased incidence of lifestyle-related diseases, such as CVDs [11,12,13] and breast and colon cancer [14,15,16,17]. These findings indicate that ingredients contained in these foods may play an important role in the inhibition of aging-associated pathologies.

Inflammation and the resulting increase in oxidative stress have been shown to contribute to most, if not all, aging-associated chronic diseases [18]. Moreover, aging itself is associated with a proinflammatory status, e.g., immune system dysregulation leading to chronic mild inflammation and sustained oxidative stress [19,20]. Chronic, low-level elevation of proinflammatory cytokines and chemokines, and the resulting increases in inflammatory biomarkers, are associated with age-related declines in function as well as increased risks of morbidity and mortality [21]. Based on this background, substances contained in foods that inhibit or counteract the aging-associated proinflammatory status and decrease resulting increases in oxidative stress (i.e., chemicals that inhibit the transfer of electrons from a substance to an oxidizing agent) have attracted scientists’ interest.

Among these substances, antioxidant polyphenols are considered to be important candidates for extending healthy lifespans. Examples include isoflavones, found at high levels in soybeans, and resveratrol, which is prevalent in the Mediterranean diet. The molecules have many biological activities that may counteract the pathogenesis of aging-associated pathologies [22,23,24]. For example, they have anti-oxidant and anti-inflammatory properties and protect cells and genes from harmful stimuli. Early animal experiments and research performed under specific conditions or in particular animals demonstrated that the increased intake of polyphenols extended lifespans [25,26]. However, the evidence from human intervention studies as well as recent animal experiments is limited, mostly inconsistent, and inconclusive, because many studies have failed to show any effects on the prevention of aging-associated pathologies and the extension of lifespan [23,27,28,29,30,31,32,33]. In addition, vitamin E and β-carotene, two anti-oxidant vitamins with potent anti-oxidant properties, increased rather than decreased the incidence of CVDs and their related mortality [34,35,36,37,38,39,40,41,42].

Chronic inflammation has been shown to be closely associated with aging-associated pathologies, and interestingly, each of these is strongly correlated with epigenetic alteration [43,44]. There is growing evidence that epigenetic mechanisms may underlie the development of aging-associated chronic diseases and may mediate the effects of nutrition. Among the factors involved in epigenetic modifications, the roles of nutrients and their metabolites on gene methylation have gathered increasing attention because methyl group donors and related molecules that contribute to gene methylation are derived from food. We have shown that aliphatic polyamines contained in foods reduce the proinflammatory status and regulate enzymatic activities involved in gene methylation and the methylation status of the entire genome [19,45]. Furthermore, experiments showed that mouse lifespans were increased by the life-time consumption of chow containing synthetic polyamines with an overall polyamine concentration of about 3 times that in soybeans [46]. Moreover, when mice with no baseline elevated risk of carcinogenesis or prior treatment with carcinogenic stimuli were fed chows with different polyamine concentrations, and then multiple, moderate doses of a carcinogen were administered, mice that were fed high-polyamine chow had a significantly lower incidence of colon tumors (mostly cancer) [47]. The current review will discuss the relation between DNA methylation and various nutrients, as well as the mechanism by which dietary polyamines affect DNA methylation and inhibit aging-associated pathologies.

## 2. Aging-Associated Changes and Immunosenescence

During the aging of an organism, there is a gradual decline of normal physiological functions. In humans, aging is associated with increased susceptibility to pathological conditions such as CVDs, sarcopenia, metabolic syndrome/diabetes, decreased kidney function, anemia, neurodegenerative diseases, cancer, and physical function impairment. The biological background of aging-associated increases in these pathologies has not yet been fully elucidated, however, it is known that environmental factors play an important role. For example, differences in aging-associated pathological changes are observed even in monozygotic twins who share the same genes.

“Inflamm-aging” is a term coined to express the close relationship between chronic inflammation and aging. Immune cells are activated when they recognize substances to be eliminated, and inflammation is generally the result of immune cell activation to eliminate harmful pathogens. A first step in this process is the binding of lymphocyte function-associated antigen 1 (LFA-1) on immune cell membranes to intercellular adhesion molecules on endothelial cells lining the innermost layer of blood vessels. The activation of immune cells results in the production of various chemical substances, including proinflammatory cytokines. Changes in immune function that are observed in the elderly are characterized by the aging-associated increase in LFA-1 expression [48,49,50,51], by shifts of immune cells and cytokine profile [52,53], and by defective humoral immunity [54]. These alterations are accompanied by progressive increases in the blood levels of proinflammatory mediators, including tumor necrosis factor α, interleukin-1, and interleukin-6 [55,56,57,58]. All three of these cytokines regulate insulin-like growth factor-1 [59], induce insulin resistance [60], inhibit erythropoiesis [61], and promote vascular dysfunction [62] and muscle wasting [63]. Their gradual increase is presumably the result of continuous stimulation by originally nonstimulatory degraded cells and other body substances [64,65].

Differences in aging-associated pathologies observed between monozygotic twins may be due to environmentally induced variations in the amounts these stimulatory materials and in the difference of immune cell response. The aging-associated pathological processes indicate the importance of reducing the aging-associated increases of substances in the body that stimulate immune cell activation, and of suppressing this activation so as to counteract the progression of aging-associated pathologies.

## 3. Polyamines

Polyamines are organic compounds that each have multiple amino groups (-NH_2_). Representative polyamines include spermidine and spermine, with three and four amino groups, respectively, and molecular weights of approximately 200 and 140 g/mol, respectively. Putrescine, a precursor of polyamine, has two amines and is therefore referred to as a diamine; its biological activities differ from those of polyamines. For example, whereas spermine and spermidine have anti-inflammatory activities and are absorbed quickly from the intestinal lumen, putrescine has minimal anti-inflammatory effects and is degraded in the intestinal lumen.

**Figure 1:**
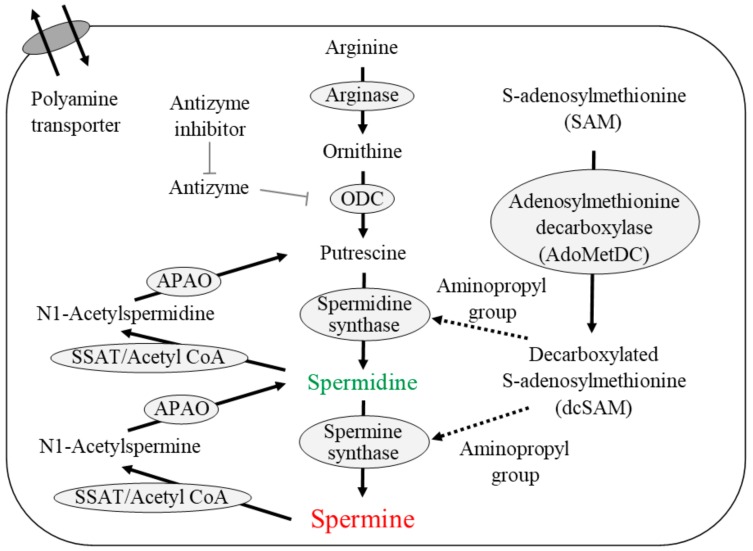
Polyamine synthesis, degradation, and transport. T-bar indicates the inhibitory activity. Arrow indicates the metabolic pathway or flow of substances. Dashed arrow indicates the supply of aminopropyl group from dcSAM. ODC: ornithine decarboxylase; SSAT: spermidine/spermine *N*^1^-acetyltransferase; APAO: *N*^1^-acetylpolyamine oxidase.

Figure 1 shows the pathway of polyamine metabolism and catabolism as well as polyamine transport. Polyamines are synthesized from arginine within cells. The activities of enzymes involved in polyamine synthesis, especially ornithine decarboxylase (ODC), decrease with aging [66,67]. ODC, a rate-limiting enzyme for polyamine synthesis, has a short half-life, and can be stimulated by various factors [66,68]. It is inhibited by antizyme, which in turn inhibited by an antizyme inhibitor. Although the properties of spermidine synthase and spermine synthase have not been fully clarified, they are considered to lack a regulatory or rate-limiting role in polyamine synthesis. The administration of arginine or ornithine stimulates putrescine levels; however, the subsequent synthesis of polyamines is not necessarily stimulated in elderly people or aged animals [67,69,70,71]. These findings indicate that the activities of spermine and spermidine synthases decrease gradually with aging. Animal tissue exhibits an aging-associated decline in ODC activity, as well as a fall in polyamine concentration [72,73].

Spermidine synthase and spermine synthase are constitutively expressed aminopropyltransferases that catalyze the transfer of the aminopropyl group from decarboxylated *S*-adenosylmethionine (dcSAM) to putrescine and spermidine to form spermidine and spermine, respectively. dcSAM is converted from *S*-adenosylmethionine (SAM) by the enzymatic activities of adenosylmethionine decarboxylase (AdoMetDC). Intracellular spermine and spermidine are degraded by spermidine/spermine *N*^1^-acetyltransferase (SSAT) and *N*^1^-acetylpolyamine oxidase (APAO). SSAT, a highly inducible enzyme, catalyzes the transfer of acetyl groups from acetyl-coenzyme A to the terminal amines of spermine and spermidine. APAO preferentially catalyzes the oxidation of the *N*^1^-acetylspermine and *N*^1^-acetylspermidine produced by SSAT activity and yields spermidine and putrescine with release of an aldehyde and hydrogen peroxide. In addition to de novo synthesis, cells can take up polyamine from the extracellular space through a polyamine transporter in the cell membrane.

Polyamines, though not putrescine, suppress the production of proinflammatory cytokines from immune cells upon stimulation with lipopolysaccharide and phorbol 12-myristate 13-acetate [74]. In addition, polyamines decrease the amount of LFA-1 on the cell membrane of immune cells [19]. The anti-inflammatory properties of polyamines are not accompanied by a decreased cellular activity. Increases in polyamine concentrations enhance the blastogenic response of immune cells to mitogens such as phytohemagglutinin and concanavalinA in vitro [19]. A very interesting finding was that in the elderly, the blastogenic response of lymphocytes to mitogens is low and the amount of LFA-1 on immune cells is high [48,51,55,75]. In addition, we have found that polyamine supplementation inhibits time-dependent decreases in the natural killer activity of immune cells obtained from peripheral blood and subsequently cultured [76]; other investigators reported that polyamines extended the duration of cultured cell activity [77].

## 4. Source of Body Polyamines

In cancer patients, extracellular polyamines have a significant effect on intracellular polyamine concentrations. Polyamine biosynthesis is up-regulated in actively growing cells, including cancer cells, and therefore, polyamine concentrations (especially spermidine concentrations) and gene expressions and activities of enzymes involved in polyamine biosynthesis are higher in cancer tissues than in normal surrounding tissues [78,79,80,81,82]. Circulating blood cells also take up polyamines synthesized in cancer cells; as a result, the blood cell concentrations and urinary excretion of polyamines, especially those of spermidine, are increased in cancer patients [78,83]. These levels decrease after tumor eradication and increase after relapse, indicating that polyamines synthesized by cancer tissues are transferred to blood cells [78].

The ability to synthesize polyamines decreases with aging. However, when polyamine concentrations in blood cells are measured in healthy human adults, the aging-associated decline in polyamine concentrations is not remarkable, and large inter-individual differences are found [19,84]. Polyamines exist in almost all living organisms, and thus, foods that are comprised of various types of organisms and their related substances contain polyamines, though at a wide variety of concentrations [85,86,87,88]. In healthy human adults, the major sources of polyamines are thought to be foods and synthesis by intestinal microbiota. Polyamines in the intestinal tract are absorbed quickly, rapidly increasing portal vein concentrations [89], and are distributed to all organs and tissues [90,91]. The exact biological mechanisms underlying the large inter-individual differences in blood polyamine concentrations in humans are not known, however, one factor is thought to be differences in the amount of polyamines supplied from the intestinal lumen, which may reflect varying preferences for foods containing polyamines. Inter-individual differences in the intestinal environment are also likely to affect polyamine synthesis possibly due to varying compositions of the intestinal bacterial flora. In fact, suppression of the polyamine supply from both foods and the intestinal microbiota results in decreased blood polyamine concentrations [92,93,94]. Conversely, a long-term increase in the polyamine supply from food gradually increases blood polyamine levels, especially spermine levels, in humans and mice [46,95]. However, such increases are not observed following short-term increases in polyamine intake; changes in diet for at least several months are required [46,95,96]. The absence of acute changes in blood polyamine levels may be due to homeostasis mechanisms that inhibit rapid alterations in intracellular polyamine concentrations.

In our latest study, in which volunteers were asked to eat fermented soybeans containing high levels of polyamines for a year, blood spermine levels gradually rose and were significantly higher than in the control group [97]. However, blood spermidine concentrations did not change, and those in the intervention group were similar to those in the control group throughout the study. Similar results were obtained in our previous animal studies and in a preliminary human trial [46,47,95]. In in vitro studies, we and others confirmed that interventions resulting in about 1.2-fold increases in spermine concentrations resulted in significant biological activity [19,98]. In our experiments, intracellular concentrations of spermidine had to increase two- to three-fold in human mononuclear blood cells to elicit apparent biological activities, i.e., suppression of LFA-1 expression and the production of proinflammatory cytokines. Among healthy volunteers, blood spermine levels inversely correlated with LFA-1 expression, while blood spermidine levels had no correlation with LFA-1 expression [19,97]. In one study involving an animal model, increased spermidine levels resulting from polyamine intake elicited favorable effects on cardiovascular physiology by activating autophagy [99]. However, increased polyamine intake by mice and humans did not increase spermidine levels in our repeated experiments in humans and mice, and many previous studies have shown that substances other than spermidine that can activate autophagy [100,101,102,103,104] did not necessarily extend lifespan or prevent CVDs [23,27,28,29,30,31,32,33]. Moreover, a study reported that in volunteers over age 90 the proportion of spermine relative to total polyamines was significantly higher than in individuals from age 60 to 80 [105]. The effects of increased polyamine intake on blood polyamine concentrations and the impact of biological activity of spermidine on physiological changes should be confirmed by other investigators.

## 5. Dietary Polyamines

All foods contain polyamines, though at widely varying levels. Therefore, personal food preferences and regional dietary patterns may greatly affect polyamine intake from food. Germ and bran, legumes such as soybeans, vegetables, and shellfish are foods with high polyamine concentrations per calorie [85,86,87,88]. The polyamine concentration in a particular food may differ depending on the part of the food examined [88,106]. For example, although fish and shellfish are lower in polyamines than beans and vegetables, higher concentrations of polyamines are found in the internal organs and roe of the fish and shellfish.

We also examined the relationship between polyamine content and dietary pattern using the food supply database of 49 Western countries from the Food and Agriculture Organization of the United Nations. The study was an ecological investigation, and the data used do not indicate the amount of foods actually consumed, however, the food supply must reflect the food demand, and thus, we examined the following relationships: the calories of specific supplied foods relative to the total calories of all supplied foods, and the amount of polyamines contained in specific supplied foods relative to the total calories in all supplied foods. This analysis of relative amounts may reflect the food preferences of the people in each country. The results of the study indicated that the Mediterranean diet was associated with an increased amount of polyamines on a per calorie basis [1,107]. A very interesting finding was that although olive oil and wine, two components of the Mediterranean diet, do not contain any polyamines, the people who preferred these foods also preferred foods rich in polyamines per calorie [107]. In contrast, people who preferred animal fat to olive oil and those who preferred spirits and beer to wine preferred foods with low polyamine concentrations [107]. In addition, people who preferred cheese (which is sometimes related to a healthy and long lifespan) also preferred foods rich in polyamines, while those who preferred milk preferred foods low in polyamines [107]. Polyamine-rich foods such as legumes, unpolished flour, vegetables, fish, and shellfish have been noted as foods relevant to a healthy long life.

Because polyamine metabolism is strictly regulated, short-term increases in polyamine intake cannot increase polyamine levels in blood cells. The finding that only long-term increases in polyamine intake can raise spermine levels in blood cells indicates that the presence of a long-lasting polyamine supply from the intestinal lumen can alter intracellular polyamine homeostasis. Changes in polyamine metabolism may affect several substances and enzymatic activities. Elevated intracellular spermine concentrations resulting from increased polyamine intake inhibits AdoMetDC activities as well as ODC, resulting in increased SAM and decreased dcSAM concentrations [108]. The increased availability of SAM enhances the activity of the methylation catalyzing enzyme DNA methyltransferase (DNMT) [109,110], and dcSAM acts to inhibit DNMT activities. These findings indicate that while polyamines have anti-oxidant and anti-inflammatory effects and protect genes and cells against harmful stimuli (Table 1), they may also affect gene methylation.

## 6. Epigenetics and Aging

A gene may be considered to be an “advanced source of digital information” comprised of combinations of four bases: adenine, guanine, thymine, and cytosine. One mechanism for regulating gene expression is DNA methylation, which is a change that involves only cytosine and creates gene information by adding a methyl group from SAM to cytosine residues at the C-5 position to yield 5-methylcytosine. Upstream of the gene, there is a direct repeat of cytosine and guanine called a CpG island. A CpG island is a site of transcription initiation, and in mammals, methylated cytosine within a CpG island can turn the gene off. Conversely, demethylation of cytosine initiates and enhances transcription, resulting in the increased production of the protein encoded by the gene.

A representative impact of diet on the function of an organism via DNA methylation is observed in the honeybee. Royal jelly affects DNA methylation patterns and causes honey bee larva to become a queen bee [152]. The importance of DNA methylation in fetal development is also observed in other animal models. Deficiencies of various vitamins, especially folic acid, often results in abnormal development. Maternal folic acid deficiency is associated with alterations of global DNA methylation and DNMT expression and activity [153], as well as with several developmental disorders [153,154,155,156]. Folate supplementation helps decrease abnormal development and regulates DNA methylation [157,158], indicating that DNA methylation plays a role in development. There have been various studies in which the importance of folate and the beneficial effect of folate supplementation on DNA methylation status and fetal development were investigated [159,160,161,162,163].

A growing number of recent studies have shown a close relationship between aging and gene methylation [164,165]. Aging is associated with enhanced demethylation of DNA in various organs and tissues in several animals and humans [166,167,168]. However, increased methylation associated with age has also been reported in some genes [169,170]. Generally, aberrant methylation status (both increases and decreased) that is associated with aging is accompanied by decreased activity of DNMT [168,171,172,173,174,175], involved in the regulation of gene methylation.

Aging-associated changes in DNA methylation status indicate that DNA methylation is susceptible to environmental stimuli. One of the most typical examples is the aging-associated differences between homozygotic twins. These twins share the same genes and they developed in the same environment. However, a considerable difference in DNA methylation pattern is observed in aged twins in comparison with young twins [176,177]. Many environmental factors have been shown to affect DNA methylation status. Various types of cellular stress seem to affect epigenetic alterations that may lead to deleterious consequences. Exposure to fine particle air pollution affects DNA methylation status in blood cells [178,179,180,181,182,183] and placenta [184]. Cigarette smoking has adverse effects on health and is associated with changes in epigenetic marks. Prenatal smoke exposure affects DNA methylation in blood cells from children of smoking mothers [185,186]. Smoking may induce DNA methylation alterations in cells of both the innate and adaptive immune systems [187]. Epigenetic alterations caused by chronic cigarette smoke sensitize bronchial epithelial cells to malignant transformation [188,189,190]. Smoking-associated changes in methylation are observed in genes related to the progression of CVDs [190,191] and the age acceleration [192,193,194]. Alcohol consumption is associated with alcohol-related DNA methylation changes in blood cells [195], and DNA methylation alterations in offspring are associated with maternal alcohol consumption [196]. Exercise alters epigenetic marks in human skeletal muscle and adipose tissue [197,198,199,200], and nutritional habits change the methylation status of the promoter area [201]. The effect of exercise on improved cardiorespiratory fitness and running performance is accompanied by widespread demethylation of CpG islands, which is the opposite of the methylation changes observed during aging [197,199]. Additionally, interventions that extended rodent lifespan inhibited age-associated changes in DNA methylation [202,203,204]. Therefore, differences in exposure to these and other environmental factors are expected to affect methylation status in homozygotic twins, resulting in differences in health conditions [205,206,207].

Alteration of methylation status with aging changes chromatin accessibility, resulting in aberrant gene transcription, as well as genomic instability. These factors may be key regulators of the aging process and contributors to the development of aging-associated diseases [208,209,210,211,212,213,214], including neoplastic growth [189,215,216,217,218,219] and aging itself [220,221,222,223]. For example, when methylation arises in the CpG islands encoding genes that suppress aging-associated disease(s) and/or when demethylation arises in the CpG islands encoding genes that cause aging-associated disease(s), the onset and the progression of aging-associated disease(s) are accelerated. Recently, aging-associated changes in multiple CpG sites across the genome in blood cells were shown to accurately predict the biological ages of individuals, independent of their chronological ages, and also predicted all-cause mortality [210,216,224,225,226,227]. This epigenetic status has been shown to be a potential biomarker of aging in humans and is associated with several aging-associated disease phenotypes [225,226,228,229,230].

## 7. Nutrients and Their Metabolites and Enzymes Related to DNA Methylation

The methylation of DNA is regulated by DNMTs (especially DNMT1, DNMT3a, and DNMT3b) in the presence of SAM, which is the methyl group donor for methylation of cytosine residues at the C-5 position that yields 5-methylcytosine. SAM, another substrate for polyamine synthesis, is converted from methionine and adenosine triphosphate (ATP) by methionine adenosyltranferase. Methionine is an essential amino acid in humans and is used in protein biosynthesis. SAM serves as a methyl group donor in many methyltransferase reactions, including DNA methylation, and is converted to *S*-adenosyl-l-homocysteine (SAH). SAH is a potent inhibitor of DNMT(s), especially DNMT1 [231,232,233], and is quickly hydrolyzed and converted to homocysteine and adenosine by adenosylhomocysteinase. Homocysteine can be recycled into methionine or converted into cysteine. Homocysteine is converted to methionine either via methionine synthase or betaine-homocysteine methyltransferase [234]. Methionine synthase requires vitamin B12 as a co-factor to transfer a methyl group from methyltetrahydrofolate to homocysteine to form *S*-methionine. Betaine homocysteine methyltransferase transfers a methyl group from betaine to form *S*-methionine. Interconversion of homocysteine and cysteine, through the intermediate cystathionine, is called the transsulfuration pathway. Cystathionine β-synthase catalyzes the conversion of homocysteine to cystathionine and water. Cystathionine is then converted to cysteine by the enzymatic activity of cystathionine γ-lyase. Vitamin B6 is required as co-enzyme by both cystathionine β-synthase and cystathionine γ-lyase.

SAM and putrescine are substrates for polyamine synthesis. An aminopropyl group is supplied by dcSAM for the synthesis of spermidine and spermine. dcSAM is converted from SAM by the enzymatic activity of AdoMetDC. dcSAM, which donates an aminopropyl group for polyamine synthesis, is a strong inhibitor of DNMT [235]. dcSAM is converted to methylthioadenosine (MTA) after donation of an aminopropyl group for polyamine synthesis. MTA is metabolized solely by MTA-phosphorylase to yield 5-methylthioribose-1-phosphate and adenine, a crucial step in the methionine and purine salvage pathways, respectively.

In humans, arginine is a semi-essential or conditionally essential amino acid, especially during the growth period. Arginine occurs at high concentrations in meats, nuts, legumes, and seafood, among others. l-ornithine, converted from l-arginine, is a non-proteinogenic amino acid that plays a role in the urea cycle and is contained in foods such as legumes and seafood. Foods with high levels of putrescine, spermidine, and spermine are described in the “dietary polyamines” section. Methionine, a substrate for SAM, is an essential amino acid and is abundant in vegetables, fruits, legumes, nuts, and meat. Homocysteine is a non-proteinogenic α-amino acid and can be recycled into methionine or converted into the amino acid cysteine with the aid of vitamin B6, vitamin B12, folate, and others. Cysteine is a semi-essential proteinogenic amino acid. Betaine participates in the conversion from homocysteine to methionine as a methyl group donor, and is contained in several foods. These facts suggest that diet has great impact on DNA methylation, and this has been proven in experimental studies. For example, high-fat, low-protein, or energy-restricted diets have been shown to be associated with and alter epigenetic marks [202,204,236,237,238,239,240].

When considering the metabolic pathway, there are two targets by which nutritional and dietary factors specifically affect DNA methylation: (1) changing the availability of methyl donors, and (2) altering the activity of DNMT by altering the concentrations of substances that inhibit DNMT.

### 7.1. Changing the Availability of Methyl Donors

**Figure 2:**
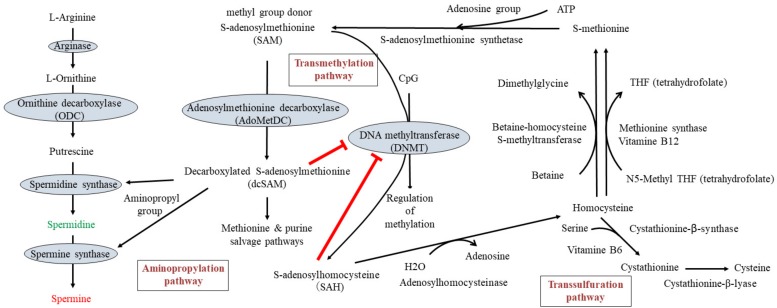
Polyamine metabolism (left), DNA methylation (middle), and one-carbon metabolism (right). T-bar indicates the inhibitory activity. Arrow indicates the metabolic pathway or flow of substance.

Recent investigations have focused on one-carbon metabolism because many dietary ingredients are involved in this metabolic pathway. Specific targets to increase the availability of methyl donor include acceleration of the conversion from homocysteine to methionine. Abnormally high levels of homocysteine have been reported to be a significant risk factor for the development of a wide range of diseases such as in cerebrovascular diseases [241,242,243,244], various CVDs [245,246,247,248,249], cognitive impairment including Alzheimer’s disease [250,251,252], fractures [253,254,255], and mortality [256]. Deficiencies of vitamin B12, folate, or vitamin B6 seem to play an important role in the occurrence of hyperhomocysteinemia, because they are essential for homocysteine metabolism (Figure 2), and treatment with B-vitamin supplementation (B6, B12, and folate) has been demonstrated to effectively lower homocysteine levels [257,258,259,260,261]. The majority of these trials have focused on the prevention of pathological changes associated with vascular dysfunction, because folic acid prevents homocysteine-induced proinflammatory status and apoptosis of endothelial cells [248,262]. A high level of homocysteine in the blood seems to provoke endothelial cell injury; this leads to inflammation in the blood vessels and accelerates atherogenesis, which can result in ischemic injury. Several studies of homocysteine-lowering treatments have shown a favorable effect on vascular pathologies associated with hyperhomocysteinemia [257,263,264]. However, other studies and meta-analyses have demonstrated that lowering homocysteine using B vitamins had no significant effect on stroke prevention [260,261,265,266], prevention of myocardial infarction [260,261,266,267,268,269,270], individual or global cognitive function [271,272,273], or other pathological conditions [274,275,276].

In addition to attenuating both inflammation and endothelial cell dysfunction [262,277], lowering homocysteine levels induces changes in the availability of methyl donors by facilitating the conversion from homocysteine to methionine, which may help regulate DNA methylation status [264,278,279,280,281]. Hyperhomocysteinemia-induced vascular pathologies, such as atherosclerosis, inflammation, hypertension, and diabetes, are also associated with alteration of DNA methylation status [282,283,284,285,286,287]. The failure of many trials of homocysteine-reducing treatments for the prevention of aging-associated diseases indicates that the alteration of methyl group availability is not sufficient to alter DNA methylation status, and thereby suppress the progression of aging-associated pathologies [288,289,290]. Similar findings have been reported in trials examining the ability of resveratrol to reduce homocysteine levels. Resveratrol significantly decreased serum levels of homocysteine in rats on a methionine-rich diet [291,292], and preferentially affected the methylation status of cancer cells [292,293,294]. However, despite extensive trials, resveratrol failed to inhibit aging-associated pathologies or extend the lifespan in mammals [27,28,29,30,31,32,33].

Based on the theoretical background of homocysteine-reducing treatment, an alternative means of affecting the availability of methyl groups is to change the amount of methionine and/or SAM intake. However, methionine supplementation in the diet specifically increases mitochondrial radical oxygen species production and mitochondrial DNA oxidative damage [277,295,296,297]. Furthermore, these biological activities of methionine provoke many deleterious effects [298,299,300]. These biological activities seem to be due to excess methionine or SAM. Although it is unknown what net effect increasing the number of methyl groups has on DNA methylation, supplementation with either methionine or SAM affects the DNA methylation status [162,298,301,302,303,304].

Conversely, methionine restriction has been shown to successfully decrease the production of mitochondrial reactive oxygen species, reduce free radical leakage, and decrease oxidative damage to mitochondrial DNA [305,306,307,308]. Furthermore, the restriction of methyl groups in foods alters DNA methylation status. Several experiments have shown that deficiency of methyl groups enhances demethylation of the entire genome and of several genes [309,310]. Other studies have shown that methionine restriction for a limited period of time, though not for long periods, affects global DNA methylation [311], inhibits chemically-induced neoplastic growth [312], and extends the lifespan of various organisms, including mammals [311,313,314,315]. One very interesting finding is that old animals fed a methionine-restricted diet demonstrated a gene methylation status similar to that of young rats [309]. These findings indicate that an important mechanism of lifespan prolongation by methionine restriction involves changes of global DNA methylation status.

### 7.2. Altering DNA Methyltransferase (DNMT) Activity

Chronic elevation in homocysteine levels results in parallel increases in SAH [316]. SAH binds with high affinity to the catalytic region of most SAM-dependent methyltransferases [233,317], enabling it to act a potent inhibitor of DNMT(s) [231,232]. The ratio of SAM to SAH, referred to as the “methylation index” has been suggested as an indicator of methylating capacity. Increased homocysteine and a low SAM/SAH ratio in plasma, both of which may reflect reduced transmethylation reactions and the resulting alteration of DNA methylation status, may be responsible for the pathogenesis of several disorders, especially angiopathy [318,319,320]. In contrast, the failure of successful vitamin treatment of hyperhomocysteinemia to decrease vascular events may be due to a weak inhibitory effect of SAH on DNMT or to the fact that vitamin treatment cannot decrease SAH levels for long enough to induce changes in the methylome and thereby prevent aging-associated pathologies.

The activity of DNMT is closely associated with the concentration of dcSAM, which is converted from SAM by the enzymatic activity of AdoMetDC, and with the dcSAM to SAM ratio [235,321]. An increase in dcSAM inhibits DNMT [321,322]. Therefore, while SAM availability regulates DNA methylation, the modulation of dcSAM activity does so more directly. Intracellular concentrations of dcSAM rise in cells in which polyamine concentrations are decreased due to several factors: decreased polyamine synthesis; overexpression of antizyme, which degrades ODC; or treatment with α-d,l-difluoromethylornithine hydrochloride (DFMO), which inhibits ODC activities [235,321,323,324]. Simultaneously, an increase in dcSAM induced by inhibition of ODC activity has been shown to decrease DNMT by decreasing DNMT protein levels and inducing hypomethylation of the whole genome [321,322,325].

**Figure 3:**
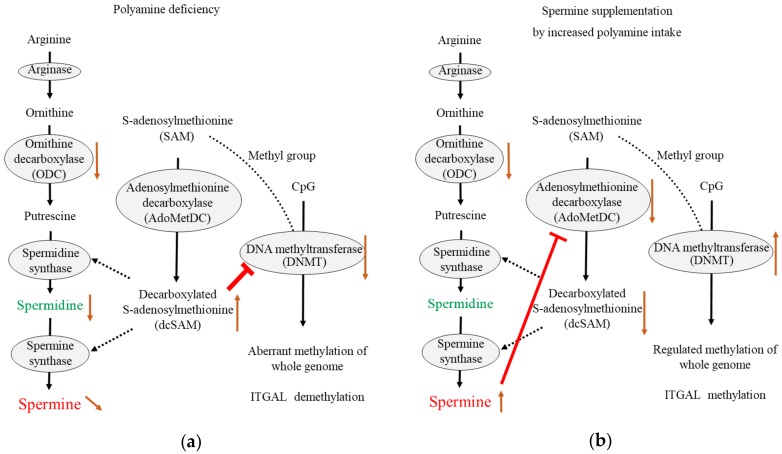
The effects of decreased ODC activity and spermine supplementation. (**a**) Decreased ODC activity increases decarboxylated *S*-adenosylmethionine (dcSAM) concentrations, because the aminopropyl group from dcSAM is not required for polyamine synthesis. Increased dcSAM inhibits DNA methyltransferase (DNMT) activity, resulting in enhanced aberrant methylation of whole genome and enhanced demethylation of ITGAL (LFA-1 promoter); (**b**) Increased spermine from extracellular sources suppresses adenosylmethionine decarboxylase (AdoMetDC) activity due to negative feedback to maintain intracellular polyamine concentrations. Decreased dcSAM concentrations induced by decreased AdoMetDC activity results in increased DNMT activity, resulting in recovery of the ability to maintain the methylation status of the entire genome and enhancement of ITGAL methylation. T-bar indicates the inhibitory activity. Black arrow indicates the metabolic pathway or flow of substances. Brown arrow indicates the increase (upward arrow) or decrease (downward arrow) of the amount of substance or the enzymatic activity.

Our latest study showed that ODC inhibition by DFMO increased dcSAM concentrations and the dcSAM/SAM ratio, and decreased activities of DNMT 1, 3a, and 3b in Jurkat cells. However, increased dcSAM concentrations did not change DNMT protein levels. In addition to ODC inhibition, spermine supplementation inhibited AdoMetDC activity and decreased dcSAM concentrations with a decreased dcSAM/SAM ratio, as well as re-activated DNMT 3a and 3b. However, DNMT 1 was not re-activated by spermine supplementation. Decreases in AdoMetDC activity and dcSAM concentrations were also achieved when Jurkat cells were supplemented with spermine alone. Similarly, changing the availability of methyl donors has been shown to affect DNMT3a and 3b expressions [326]. A methylation microarray was used to analyze the effects of DFMO and spermine on the methylation status of the entire genome. The restriction enzyme *NotI* cleaves a specific DNA sequence, however, when cytosine in the sequence is methylated, this cleavage fails. Microarray analyses of the methylome at the site of *NotI* cleavage showed that increased dcSAM with decreased polyamine concentrations were associated with aberrant methylation of the entire genome. Depending on the portion of the genome, the decline in DNMT activity not only induced increases in genome demethylation but also reinforced the methylation at other locations. In other words, polyamine deficiency both increased demethylation in certain areas and increased methylation in other areas [45,47]. Conversely, decreases in dcSAM concentrations induced by spermine supplementation were associated with suppression of aberrant methylation induced by ODC inhibition (Figure 3).

## 8. Aging, Polyamines, and DNA Methylation

Generally, aging is associated with decreases in ODC [327] and DNMT activities [174,328,329], and enhanced demethylation of the LFA-1 promoter area in association with increases in LFA-1 protein levels [19,97,330]. Decreases in DNMT activity decrease donations of methyl groups to cytosine residues, and seem to enhance genome-wide demethylation; however, DNA methylation drift seems to be a non-directional change as it involves both hypermethylation and hypomethylation events during aging [47,177,331,332,333,334,335]. In a murine model involving chows with different polyamine concentrations, the methylation status of the entire genome in old mice fed regular or low-polyamine chow showed an increase in aberrant methylation. However, lifelong intake of high-polyamine chow prevented aging-associated increase in aberrant methylation [47]. The regulation of methylation status by polyamine intake was very similar to that observed in our in vitro study in which DNMT suppression resulting from DFMO-induced ODC inhibition caused aberrant methylation, while spermine supplementation reversed this condition [45].

Bi-directional changes in methylation status were also observed in the promoter area of LFA-1 (called ITGAL). Detailed base sequencing after treatment with bisulfite, which converts unmethylated, though not methylated, cytosine to uracil, showed that the site responsible for LFA-1 expression in immune cells [165,336] was demethylated and associated with increased LFA-1 protein levels after ODC inhibition. However, other CpG sites in ITGAL were either demethylated or methylated in a site-specific manner. Spermine supplementation reversed spermine deficiency-induced demethylation of the CpG area responsible for LFA-1 expression and decreased LFA-1 protein levels. Similarly, changes in the status of DFMO-induced methylation in most other areas were almost reversed by spermine supplementation. DNMT and SAM seem to act together to regulate methylation status, and defective functional activity of DNMT may fail to maintain appropriate methylation [337].

Since polyamines have many biological activities that may counteract aging-associated pathologies, and play an important role in the regulation of DNA methylation, my colleagues and I hypothesized that increased polyamine intake may reduce the incidence of conditions linked to old age. We demonstrated that lifelong consumption of polyamine-rich chow increased blood spermine levels, inhibited aging-associated pathological changes in mouse organs, inhibited aging-associated increases in LFA-1 protein levels, and extended mouse lifespans [46,47,95]. In our latest human interventional trial, increased polyamine intake for 1 year increased blood spermine levels and inhibited aging-associated increases in LFA-1 protein levels [97].

## 9. Possible Role of Polyamines in Inhibiting Tumorigenesis

Aging is one of the largest risk factors of carcinogenesis [338], and there is growing evidence that aging-associated changes in DNA methylation status are closely related to the occurrence of cancer [339,340,341,342]. Given the many biological activities of polyamines (Table 1) that may inhibit aging-associated pathological processes, including the progression of aberrant DNA methylation, and the experimental findings showing that increased polyamine intake extended animal lifespans, it is reasonable to assume that increased polyamine intake may suppress neoplastic diseases. To test this hypothesis, animal models were employed in which dietary patterns and carcinogen exposure were similar to those in humans. The majority of humans are born without an increased risk of tumorigenesis and grow up with a regional dietary pattern. Under such circumstances, humans are exposed to weak, though repeated carcinogenic stimuli throughout their lives. BALB/c mice were fed chows with different polyamine concentrations, and were then repeatedly administered moderate amounts of a carcinogen (20 mg/kgBW of 1,2-demethylhydrazine once a week for 12 consecutive weeks). Mice fed high-polyamine chow had a lower incidence of neoplastic growth (mostly colon cancer) [47]. Similar results were obtained by a group at Josai University, which examined enhancement of tumorigenesis by increased polyamine intake. However, in rats administered low-dose (85 mg/kgBW) 2-amino-1-methyl-6-phenylimidazole for 8 days and chows containing three different polyamine concentrations, increased polyamine intake did not increase carcinogenesis and even seemed to suppress it [343].

ODC is a focus of therapies that aim to prevent carcinogenesis because it is a transcriptional target of a proto-oncogene [344,345,346], and many studies have shown that transfection of the ODC gene results in increased intracellular polyamine levels and malignant transformation [347,348,349,350,351,352,353]. Most reports on the roles of ODC in malignant transformation have examined cells that were already at risk of tumorigenesis. In contrast, malignant transformation after transfection of the ODC gene in normal cells has not been observed [354,355,356]. Reaffirming the importance of ODC inhibition in tumorigenesis, increased polyamine concentration reduces ODC translation in reticulocyte lysates [357,358] and in cell cultures [359]. In cells with normal homeostasis, the influx of polyamines from the extracellular space suppresses ODC activity, with spermine being the most effective, and putrescine the least effective polyamine in regulating ODC activity [98]. One previous study reported significant suppression of ODC activity in the intestinal mucosa of rats fed chow with high polyamine concentrations [96].

In a cohort study, increased polyamine intake from food was associated with an increased number of colon polyps in patients who were already at high risk [360]. However, a study of subjects at low risk of neoplastic diseases showed that increased polyamine intake was associated with decreased tumorigenesis (colon polyps) [361]. These results are similar to those in animal studies, in which increased polyamine intake accelerated tumorigenesis in animals at high risk of tumorigenesis, while increased polyamine intake followed by repeated weak carcinogenic stimuli decreased tumorigenesis. In addition, a polyamine-rich diet [88,107] was associated with a decreased incidence of breast and colon cancer [14,15,16,17].

## 10. Future Perspectives

**Figure 4:**
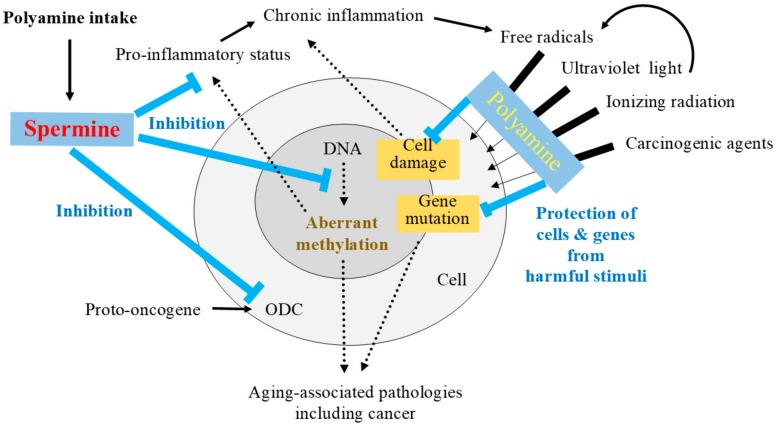
The role of spermine in the inhibition of aging-associated pathologies. Increased polyamine intake elevates spermine levels. Spermine is converted to spermidine by spermidine/spermine *N*^1^-acetyltransferase (SSAT) and *N*^1^-acetylpolyamine oxidase (APAO). Increased polyamines (spermine and spermidine) can protect cells and genes from harmful stimuli. Additionally, increased spermine activates the negative feedback system to inhibit the activities of ODC and AdoMetDC. ODC is a transcriptional target of a proto-oncogene, and AdoMetDC inhibits DNMT activity by increasing dcSAM concentrations, and thus, regulating the methylation status of DNA. Decreased damage of cells and genes as a result of polyamine activity inhibits aging-associated increase in originally inoffensive substances that provoke chronic immune cell activation. Spermine-induced suppression of the pro-inflammatory status observed with aging (e.g., increased production of pro-inflammatory cytokines, and increased expression of lymphocyte function-associated antigen 1 (LFA-1) on immune cells) may help inhibit the progression of aging-associated diseases. T-bar indicates the inhibitory activity. Black arrow defines the relation of upstream and downstream. Change in thickness of arrows indicates the barrier effect of polyamine from harmful stimuli.

The biological activities of polyamines that may help inhibit aging-associated pathologies are summarized in Figure 4. At this point, despite extensive studies, there is no proof that changes in specific DNA methylation patterns, in a specific signal transduction pathways, or specific proteins can extend lifespans, especially in mammals [213]. Instead, there is an overwhelming scientific consensus supporting the important role of epigenetic changes in aging-associated pathologies and lifespan alteration [173,210,226,362,363,364,365,366,367]. Based on these perspectives, it is important to maintain levels of DNMT, which decrease with age and inhibit aging-associated aberrant DNA methylation. This requires sustained biological activity, because many environmental factors continuously interfere with the gene methylation status.

Sustained elevations in polyamine concentrations by food intake, and the resulting changes in intracellular homeostasis, must continuously regulate the methylation status of various genes relevant to the onset and progression or the inhibition of aging-associated diseases. From the point of view of chronic inflammation, the ability of polyamines to prevent noxious stimuli from damaging genes, cells, and tissues can inhibit aging-associated increases in the production of compounds from originally inoffensive substances in the body. Unlike viruses, bacteria, and other pathogenic microorganisms that strongly arouse immune activation, such compounds weakly stimulate immune cell activation. In addition to the possible reduction of these compounds, spermine suppresses LFA-1 expression via regulation of gene methylation. Because inflammation is also closely associated with gene methylation [44,368,369,370,371], suppression of immune cell activation may counteract the progression of aging-associated pathologies.

The difference in the effects of spermine and those of aging on the methylation of individual genes has not been fully elucidated. In addition, the effect of spermine on DNMT levels and activity should be further investigated. In an experiment on the effects of aging and spermine on LFA-1 expression, subsets of immune cells in which expression is increased with aging seemed to be different from those in which LFA-1 is decreased by spermine [19,97]. These results indicate that spermine-induced changes in methylation status in genes and various cell types must not be the same as those induced by aging. Elucidating these differences and developing approaches to increase blood spermine levels are crucial for extending the human lifespan.

## Figures and Tables

**Table 1:** Biological activity of polyamines.

Activity	Authors	Journal (Year)
Anti-inflammation	Lovaas E. et al.	*Free*. *Radic*. *Biol*. *Med*. 1991, *11*, 455–461. [111]
	Zhang M. et al.	*J*. *Exp*. *Med*. 1997, *185*, 1759–1768. [74]
	Soda K. et al.	*J*. *Immunol*. 2005, *175*, 237–245. [19]
	Lagishetty C.V. et al.	*Indian*. *J*. *Pharmacol*. 2008, *40*, 121–125. [112]
	Choi Y.H. et al.	*J*. *Biomed*. *Sci*. 2012, *19*, 31. [113]
	Paul S. et al.	*Inflamm*. *Res*. 2013, *62*, 681–688. [114]
	Zhou S. et al.	*Front*. *Immunol*. 2018, *9*, 948. [115]
Anti-oxidant & Free radical scavenger	Tadolini B. et al.	*Biochem*. *Biophys*. *Res*. *Commun*. 1984, *122*, 550–555. [116]
	Lovaas E. et al.	*Free Radic*. *Biol*. *Med*. 1991, *11*, 455–461. [111]
	Khan A.U. et al.	*Proc*. *Natl*. *Acad*. *Sci*. *USA* 1992, *89*, 11428–11430. [117]
	Goss S.P. et al.	*Chem*. *Res*. *Toxicol*. 1995, *8*, 800–806. [118]
	Marzabadi M.R. et al.	*Free*. *Radic*. *Biol*. *Med*. 1996, *21*, 375–381. [119]
	Farbiszewski R. et al.	*Neurochem*. *Res*. 1996, *21*, 1497–1503. [120]
	Ha H.C. et al.	*Proc*. *Natl*. *Acad*. *Sci*. *USA* 1998, *95*, 11140–11145. [121]
	Jung I.L. et al.	*Arch*. *Biochem*. *Biophys.* 2003, *418*, 125–132. [122]
	Chattopadhyay M.K. et al.	*Proc*. *Natl*. *Acad*. *Sci*. *USA* 2003, *100*, 2261–2265. [123]
	Belle N.A. et al.	*Brain Res*. 2004, *1008*, 245–251. [124]
	Gaboriau F. et al.	*Redox*. *Rep*. 2005, *10*, 9–18. [125]
	Fujisawa S. et al.	*Anticancer Res*. 2005, *25*, 965–969. [126]
	Sava I.G. et al.	*Free Radic*. *Biol*. *Med*. 2006, *41*, 1272–1281. [127]
	Rider J.E. et al.	*Amino*. *Acids* 2007, *33*, 231–240. [128]
	Nayvelt I. et al.	*Biomacromolecules* 2010, *11*, 97–105. [129]
	Jeong J.W. et al.	*Biomol*. *Ther*. *(Seoul)* 2018, *26*, 146–156. [130]
Radioprotection	Courdi A. et al.	*Int*. *J*. *Cancer* 1986, *38*, 103–107. [131]
	Arundel C.M. et al.	*Radiat*. *Res*. 1988, *114*, 634–640. [132]
	Held K.D. et al.	*Int*. *J*. *Radiat*. *Biol*. 1991, *59*, 699–710. [133]
	Snyder R.D. et al.	*Radiat*. *Res*. 1994, *137*, 67–75. [134]
	Williams J.R. et al.	*Biochem*. *Biophys*. *Res*. *Commun*. 1994, *201*, 1–7. [135]
	Spotheim-Maurizot M. et al.	*Int*. *J. Radiat*. *Biol*. 1995, *68*, 571–577. [136]
	Newton G.L. et al.	*Radiat*. *Res*. 1996, *145*, 776–780. [137]
	Chiu S. et al.	*Radiat*. *Res*. 1998, *149*, 543–549. [138]
	Sy D. et al.	*Int*. *J*. *Radiat*. *Biol*. 1999, *75*, 953–961. [139]
	Warters R.L. et al.	*Radiat*. *Res*. 1999, *151*, 354–362. [140]
	Douki T. et al.	*Radiat*. *Res*. 2000, *153*, 29–35. [141]
	von Deutsch A.W. et al.	*Gravit*. *Space Biol*. *Bull*. 2005, *18*, 109–110. [142]
Protection from ultraviolet light	Snyder R.D. et al.	*Photochem*. *Photobiol*. 1990, *52*, 525–532. [143]
	Williams J.R. et al.	*Biochem*. *Biophys*. *Res*. *Commun*. 1994, *201*, 1–7. [144]
	Pothipongsa A. et al.	*Appl*. *Biochem*. *Biotechnol*. 2012, *168*, 1476–1488. [145]
Protection from chemicals & other stress	Rajalakshmi S. et al.	*Biochemistry*. 1978, *17*, 4515–4518. [144]
	Mackintosh C.A. et al.	*Biochem*. *J*. 2000, *351*, 439–447. [146]
	Di Mascio P. et al.	*Arch*. *Biochem*. *Biophys*. 2000, *373*, 368–374. [147]
	Chauhan S.D. et al.	*FASEB*. *J*. 2003, *17*, 773–775. [148]
	Gugliucci A. et al.	*Life Sci*. 2003, *72*, 2603–2616. [149]
	Sagor G.H. et al.	*Transgenic Res*. 2013, *22*, 595–605. [150]
	Okumura S. et al.	*Liver Transpl*. 2016, *22*, 1231–1244. [151]

## Abbreviations

DNMT	DNA methyltransferase
SAM	*S*-adenosylmethionine
SAH	*S*-adenosyl-l-homocysteine
dcSAM	decarboxylated *S*-adenosylmethionine
CVD	cardiovascular disease
LFA-1	lymphocyte function-associated antigen 1
ODC	ornithine decarboxylase
AdoMetDC	adenosylmethionine decarboxylase
SSAT	spermidine/spermine *N*^1^-acetyltransferase
APAO	*N*^1^-acetylpolyamine oxidase
MTA	methylthioadenosine
DFMO	α-d,l-difluoromethylornithine hydrochloride
